# Long-Term Esthetically Depigmented Gingiva in a Short Operative Duration, Using Two Modes of 940 nm Diode Lasers—A Randomized Clinical Trial

**DOI:** 10.1155/2022/8215348

**Published:** 2022-11-24

**Authors:** Baydaa F. Hamzah, Abrar N. Alattar, Tiba A. Salman

**Affiliations:** ^1^Department of Oral Surgery and Periodontology, College of Dentistry, Mustansiriyah University, Baghdad, Iraq; ^2^Oral and Maxillofacial Surgery, Department of Oral Surgery and Periodontology, College of Dentistry, Mustansiriyah University, Baghdad, Iraq; ^3^Department of Prosthodontic, College of Dentistry, Mustansiriyah University, Baghdad, Iraq

## Abstract

**Aim:**

This study aims to compare the effectiveness of the two modes of 940 nm diode lasers on gingival depigmentation.

**Methods:**

Twenty patients (11 females and 9 males) participated in this study; they were free of any systemic or local condition and randomly assembled into two groups: continuous wave (CW) and pulsed groups, in contact modes. The diode laser was of 940 nm wavelength and 1-2 W irradiation power for the two modes in all cases. A single session of irradiation was applied to the facial gingivae of the upper and lower anterior segments. Photographs were taken preoperatively and postoperatively for all patients, and a questionnaire and follow-up were carried out for the next four weeks and after three years.

**Results:**

The intraoperative duration of both modes was considerably short, with a short chair time subsequently. A statistically significant decrease in the intensity of pain, discomfort, and bleeding (*p* ≤ 0.05) was noticed during the first week of follow-up within each group. Nonetheless, the two groups showed a close pattern of decline with no statistically significant differences between them. It was also clear that pain and discomfort were strongly correlated with each other within each group, and the correlation was statistically significant. On the other hand, carbonization was minimal in a few cases treated with CW mode but still not statistically significant. Finally, although the gingival pigmentation index (GPI) differences were not statistically significant between the two groups, they were significant within each group.

**Conclusions:**

The diode laser (940 nm) offered an effective and safe modality, providing an optimal aesthetic result that can meet patient satisfaction with minimal pain, bleeding, discomfort, intraoperative time, and the possibility of recurrence in the treatment of gingival hyperpigmentation.

## 1. Introduction

Melanin is a brown pigment that appears as a result of melanin granules produced by melanocytes of the basal and suprabasal layers of the gingival epithelium [1, 2]. It is a natural pigment that contributes to the predominantly endogenous pigmentation of gingiva and oral mucosa [2]. This natural pigmentation arises as an aesthetic complaint that requires treatment, mainly if it is noticeable during speech or smiling, as seen in patients with gummy smiles [3, 4].

The management of gingival depigmentation involves different therapeutic approaches, among which the conventional scalpel technique, free gingival autograft or cellular dermal matrix allograft to conceal pigmented gingival, abrasion with a large round diamond bur, and lasers [5].

Nowadays, the laser has been distinguished as the most effective, reliable, and comfortable technique for the ablation of cells that contain and produce melanin pigment. It is less invasive with minimum postoperative bleeding and pain when compared to the most commonly used conventional scalpel technique, in which several drawbacks were documented, such as healing by secondary intention, experiencing pain, discomfort, and unpleasant bleeding both intraoperatively and postoperatively, and a subsequent extended chair time added to the necessity of the periodontal pack protection and the delicate scar left at the surgical sites [5–7].

On the other hand, the laser technique has many advantages, among which are the following:

1. Reduced mechanical trauma, postoperative pain, swelling, and scarring;
2. Surgical site sterilization via bactericidal effects;
3. Ability to coagulate, vaporize, and cut tissues;
4. Dry surgical field as a result of homeostasis following diode laser irradiation that seals off the adjacent blood vessels in the surrounding tissue up to a diameter of 0.5 mm; and
5. Decreased intraoperative time, all of which are helpful, particularly during the current circumstances of the COVID-19 pandemic. Whereas the most commonly encountered disadvantage of lasers is their relatively high cost [6, 8, 9].

The latest literature has documented evidence of successful depigmentation using diode lasers, and considered it as the best laser for soft tissue treatment due to the smaller size of the unit and lower cost if compared to other types of lasers, added to shallow tissue penetration that prevents deleterious effects to the teeth roots [4, 10–12]. Nevertheless, the process of gingival depigmentation should be performed carefully with adjacent teeth protection, since an improper laser application may postpone wound healing and cause gingival collapse, as well as underlying tissue damage of the periosteum and bone [13]. Other types of lasers used in gingival depigmentation are Nd: YAG, Er: YAG, and CO_2_ lasers [14].

This study compared the effectiveness of two different modes of 940 nm diode laser on gingival depigmentation since earlier studies have only compared different types and/or wavelengths of the same laser mode.

## 2. Materials and Methods

The Human “Research Ethical Committee” at the College of Dentistry/University of Mustansiriyah/Baghdad, Iraq, approved the ethical standards in this study, in compliance with the Declaration of Helsinki, 1975. With a reference number (REC109).

The study was conducted on 20 eligible patients who attended the College of the Dentistry/University of Mustansiriyah seeking aesthetic management of hyper-pigmented gingiva anteriorly and were willing to participate in the study.

Participants were free from any systemic or local condition that might affect the healing process, such as autoimmune diseases, uncontrolled diabetes, smoking, pregnancy, or periodontal diseases. The consort flow chart of eligibility, allocation, and analysis is presented in (Appendix Figure 1).

Written informed consent was obtained from all patients after explaining the procedure`s details and the study's purpose.

The patient sample size was determined in accordance with previous studies [15, 16] and included 11 females and 9 males, whose mean ages ranged from (16–29) years. They were divided randomly in a parallel manner into two groups; the first ten participants were included in the continuous wave (CW) group and treated by CW laser mode, and the other ten were treated by pulsed laser mode (cp1) in the pulsed group; all of them were blinded to the assignment.

### 2.1. Surgical Procedure

Operating staff and patients wore protective eyeglasses, particularly designed for diode lasers at 940 nm (diode Epic, BioLase, USA), to fulfill the FDA laser safety rules. Reflective instruments were not used during the surgical procedure to avoid laser beam reflection, and the area surrounding the surgical site was protected with sterile gauze. Treatment was achieved in a single session and was painless, as stated by the participants, with no need for any type of local anesthetic [4].

The 940 nm wavelength diode laser was emitted in continuous mode for the CW group and in pulsed mode for the pulsed group by a 400 nm diameter flexible fiber optic hand-piece tip that is initiated with a special block, and the aiming beam was 625 nm. The power setting was gradually increased in both groups, from 1– 2 W, according to the operator's experience and manufacturer instructions.

Laser ablation was achieved in a brushing or painting-like technique beginning from the mucogingival junction toward the free gingival margin, from the second premolar to the contralateral tooth of the jaw; whenever the interdental papilla was hyperpigmented it was also included in the ablation process.

During the procedure, the epithelial remnants after laser ablation were removed with sterile gauze soaked in normal saline to enhance visualization and cool the tissue until the gingival surface appeared free of pigmentation. Thus, enough care was taken to lessen the laser's thermal side effects on the surgical site in order to avoid unnecessary tissue necrosis after laser application.

The laser ablated the gingiva and gradually removed the pigment without any bleeding, though some bleeding may be noticed during the ablation. Consequently, no periodontal pack was needed.

Some deep black or brown spots that do not disappear during the procedure were left to avoid overheating or bone exposure. At the end of the appointment, each patient was given a personal evaluation questionnaire to be answered for clinical analysis. Clinical photographs were taken during the preoperative, immediate postoperative, succeeding four weeks, and after 3 years of the surgery, as displayed in (Appendix Figures 2 and 3).

### 2.2. Effectiveness Assessment

#### 2.2.1. Clinical Effectiveness Parameters

The two modes were compared via clinical parameters: pain, discomfort, bleeding, and intraoperative duration. Pain and discomfort were evaluated postoperatively after the 1st, 2nd, 3rd, and 4th weeks, whereas bleeding was assessed immediately postoperatively and for the following two weeks.

The degree of pain was indexed using the visual analogue scale (VAS), a 10 cm horizontal, continuous interval scale with its left endpoint marked as “no pain” and its right endpoint marked as “severe pain.” The patients checked a mark to evaluate their postsurgical pain level on each day of the first week by answering a questionnaire specially designed for this research [17].

Scores of pain were measured as follows:

  0 = No pain;
  0.1–3.0 cm = Slight pain;
  3.1–6.0 cm = Moderate pain;
  6.1–10.0 cm = Severe pain.

A similar scale was used to evaluate the degree of patients' discomfort.

Bleeding was assessed immediately after surgery and for the next two weeks. Bleeding scores were measured as follows: [18].

*A* = None; *B* = Slight; *C* = Moderate; *D* = Severe.

#### 2.2.2. Perceptual Effectiveness Parameters

Melanin pigmentation in the oral cavity is classified differently according to its color degree, gingival position, and gingival color alteration [19]. In this study, preoperative assessment of each case was achieved using the GPI (gingival pigmentation index), in which scores were as follows [20]:

  0 = absence of pigmentation.
  1 = spots of brown to black color or pigments.
  2 = brown to black patches but not diffused pigmentation.
  3 = diffused brown to black pigmentation, marginal, and attached.

Then, the GPI scores of the two groups were evaluated within the first month and after 3 years of the treatment to assess its effectiveness and adequacy to fulfill patients` satisfaction.

### 2.3. Statistical Analysis

Data analysis was performed using statistical software (SPSS version 20 for Windows, France). Since it is nonparametric ordinal data, nonparametric tests were applied. Mann—Whitney *U* test was used to identify the statistical differences in GPI, operative duration, post-operative pain, discomfort, and bleeding between the two groups, whereas the Friedman test was used to explore the statistical differences of the same variables within each group, with a level of *p* ≤ 0.05 considered to be statistically significant. The Spearman correlation coefficient was applied to check the correlations of parameters to each other.

## 3. Results

In this study, twenty patients were evaluated, with their mean age ranges ± SD being 21 ± 4.7 years in the CW group versus 23 ± 3.9 years in the pulsed group. The demographic data are detailed in (Table 1), with no statistically significant difference between the two groups.

### 3.1. Comparison of the Operative Duration

A very slight difference was noticed between the two groups regarding mean operative duration ± SD. Results were 3.5 ± 1.6 minutes and 3.7 ± 1.7 minutes for CW and pulsed groups successively, which were not statistically significant as well (*p* ≤ 0.79).

### 3.2. Pain Differences

**Figure 1:**
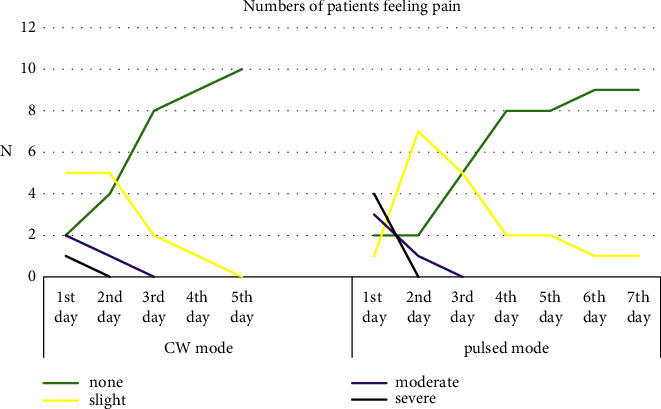
Reveals a decreased number of patients feeling pain during the first week of treatment with either CW or pulsed diode laser modes.

One patient in the CW group stated severe pain on the first day postoperatively, whereas five patients out of 10 (50%) complained of slight pain on the 1st and 2nd days, and by the 5th day, 100% of the CW group participants recorded no pain. However, in the pulsed group, 40% of patients (4 out of 10) had severe pain on the 1st day, and 70% of them had slight pain on the 2nd day, after which the percentage of patients feeling pain gradually decreased till it reached 10% at the end of the first week. Mann—Whitney *U* test was applied to compare the pain VAS scores between the two groups; the differences were statistically not significant, with a gradual decrease of pain sensation among patients in both groups throughout the first week of the follow-up period, as displayed in (Figure 1). This decrease was significant statistically within each group (*p* ≤ 0.05).

### 3.3. Discomfort Variations

**Figure 2:**
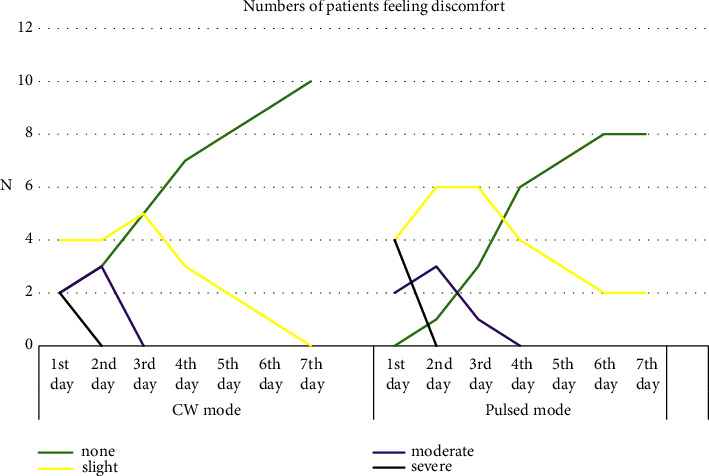
Reveals a decreased number of patients feeling discomfort during the first week of treatment with either CW or pulsed diode laser modes.

In the CW group, nearly half of the patients (40%) recorded mild discomfort on the first 3 days postoperatively, and all of them revealed no discomfort by the 7th day. Likewise, in the pulsed group, 40% of patients had mild discomfort on the 1st day, which increased to 60% on the next two days and then decreased to 20% by the end of the first week. Again, the differences between the two groups were statistically not significant. As shown in (Figure 2)

Yet, the decrease in discomfort sensation was statistically significant within each group throughout the first week (*p* ≤ 0.05).

### 3.4. Bleeding Tendency

Clinically, bleeding neither occurred postoperatively nor was mentioned by the patients in the succeeding two weeks of follow-up, except for 30% of the CW group participants who had slight bleeding immediately after surgery. Again, the differences between the two groups were statistically not significant (*P* ≤ 0.067), as shown in (Table 2).

### 3.5. Correlations of Pain and Discomfort Patterns

An approximately similar statistically significant pattern of pain-discomfort correlations was observed in both CW and pulsed groups, for the first group; pain and discomfort of the first 2 days exhibited very strong positive correlation values (spearman rho = 0.936, 0.795, *p* ≤ 0.000, 0.006) successively. Where pain on the second day correlated strongly with that on the first day (rho = 0.777, *p* ≤ 0.008), this correlation was also true for discomfort on each pair of succeeding days, i.e., the 1st with the 2nd, the 3rd with the 4th, etc.

Yet, for the pulsed group, pain on the 1st day was strongly related to discomfort on the first 3 days, and pain on days 2–4 was strongly and directly proportionate as well, whereas discomfort followed the same pattern as the CW group, with a very strong positive correlation at the 4th with 5th days (rho = 0.802, *p* ≤ 0.005) and a strong positive correlation for the 2nd with the 3rd, the 5th with the 6th, and the latter with the 7th days.

### 3.6. Gingival Pigmentation Index (GPI) Changes

**Figure 3:**
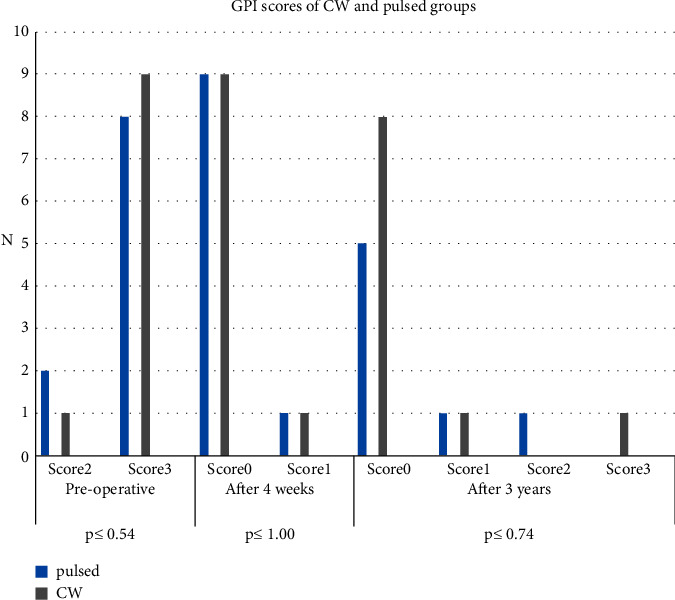
The GPI scores of the CW and pulsed groups at three follow-up time intervals.

Mann—Whitney *U* test was applied to compare the GPI scores of the two groups, and it revealed no statistically significant differences between the two modes. Meanwhile, the changes within each group were statistically significant among the surveyed three-time intervals, excluding three cases of lost 3-yearfollow-up in the pulsed group, as shown in (Figure 3).

## 4. Discussion

Currently, due to its advantages, gingival depigmentation by laser is identified as the treatment of choice among clinicians [16]. The diode lasers can be applied for depigmentation in dentistry since they are of the 800–980 nm wavelength spectrum [21, 22], which are highly absorbed and lie within the absorption spectrum of pigments, like melanin, which ranges between 351 and 1064 nm [22, 23], as well as hemoglobin and other pigments.

When compared to a scalpel, a diode laser introduced many advantages related to decreased treatment chair time along with postsurgical pain and discomfort [10, 24–26], added to the absence of painful injections [27]. These advantages increased patients' acceptability and convenience about this treatment modality, including the participants in this study.

A standard characteristic of laser wound healing during the first several days is the white fibrin slough, which appeared in almost all patients in the current study after 24 hours. This might be due to the comparatively thick protein coagulation layer on the treated area as a result of the diode laser fiber optic effect “hot tip.” This layer serves as a biological wound dressing to seal the ends of sensory nerves [10, 28]. Though on the day of treatment, the pain was reported and some participants described slight, moderate, or sometimes severe pain and discomfort during acidic, salty, or spicy foods consumption because they didn`t comply with the operator`s postoperative instructions, which was also reported by Şimşek Kaya et al. [16].

However, pain disappeared completely by the 5th day following the CW mode application in this study, but it took two more days to disappear in the CW group of Jananni et al.'s study [29].

The discomfort was proportional to the pain in both groups, especially in the early postoperative days, with a strong statistically significant correlation and a continuous decrease in these two parameters. This reduction of pain and discomfort intensity might be due to the minimal thermal damage to the adjacent tissue since diode laser penetrates less than other types of lasers, minimizing the harmful effects on the teeth roots [29].

After one week of the treatment session in this study, the appearance of gingival tissue was clinically normal with complete epithelization in both groups. The fast clinical healing was supported by the statistically significant decrease in pain and discomfort perception in both groups within the first week. The healing period in the current study was less than the 3-month healing interval obtained by Elemek after depigmentation with an 810 nm diode laser [30] and that of Esen et al., who reported a complete cure within 2 weeks after depigmentation with a CO_2_ laser in super-pulse mode at 10 W [31]. On the other hand, Atsawasuwan et al. noticed that healing was obvious three to four weeks after depigmentation using Nd: YAG laser pulses at 6 W [3].

In this study, no patient complained of visible swelling postoperatively; this is in line with Khakhar et al., who attributed complete removal of the gingival epithelium with no microvascular dilatation to the direct vasomotor effects and/or local proinflammatory mediator's deactivation by diode laser light, leaving narrowed microvessels [32].

Bakutra et al. noticed that 90% of cases showed slight bleeding immediately after surgery [26]. Nonetheless, the CW group in the current study showed slight bleeding only in 30% of patients immediately after the treatment session, which is close to El Shenawyet al. results, where only 20% of cases bled slightly [17]. On the other hand, the pulsed mode group of this study revealed the absolute absence of bleeding, which is probably due to deeper penetration of the CW beam affecting connective tissue [30], along with laser absorption by soft tissue pigments that promotes a perfect haemostatic agent. [27] Furthermore, during the 1st and 2nd weeks of follow-up, no bleeding was recorded by any patient in both group; this is in line with Bakutra et al. [26] and El Shenawy et al. [17] that showed no sign of bleeding within the first week of the treatment.

The laser depigmentation parameters are established based on the primary effect of melanin removal as well as the repigmentation tendency, bearing in mind that the mechanism of the latter may be either due to high melanocyte activity or to incomplete ablation of these cells with subsequent migration from the adjacent free gingiva to the depigmented sites [33].

The three-time intervals of follow-up in this study represented statistically significant GPI changes within each group and indicated promising final aesthetic results. All participants were satisfied with the results of the treatment, due to the massive clinical changes in their gingival aesthetic appearance and consequently quality of life that is seen throughout the relatively long follow-up period.

Despite the two cases of repigmentation in which participants declared that they smoked afterwards that can justify the recurrence [34].

Nevertheless, repigmentation is common and documented differently depending on the treatment modality and follow-up period. In the current study, repigmentation was observed in two out of twenty cases (10%). An exactly similar result was documented by Singh et al. in their 18-month of comparative observational period after depigmentation with diode laser and cryosurgery [27]. It is also close to the result reported by Esen et al., in which two out of ten (20%) patients showed recurrence after ablation of hyper-pigmented gingiva using a CO_2_ laser through 12–24 months of follow-up period postoperatively [31].

On the other hand, Nakamura et al. documented repigmentation in four out seven cases within two years of follow-up after CO_2_ laser gingival depigmentation [35], whereas other patients noticed no recurrence within 6 months of the follow-up period [7].

## 5. Conclusion

To conclude, despite the limitations of this study, CW and pulsed modes of the 940 nm diode laser were observed to be equally effective and safe to enhance optimal aesthetic results and to reduce pain, discomfort, and bleeding, as well as the intraoperative duration of gingival depigmentation, with a lower possibility of recurrence over 3 years of follow-up, when compared to other types of lasers.

## Figures and Tables

**Table 1:** Displays the demographic data of participants in both groups.

Independent variables		CW	Pulsed	*P* value
		*N*	*N*	
Gender	Female	3	8	0.07
	Male	7	2	

Age groups (years)	<20	5	2	0.34
	20–24	2	4	
	25+	3	4	

Jaw	Mandible	1	3	0.58
	Maxilla	9	7	

**Table 2:** Displays the differences in bleeding availability between CW and pulsed groups using the Mann–Whitney *U* test with their frequencies (*N*) and percentages (%).

		CW mode *N* (%)	Pulsed mode *N* (%)	*P* value
Bleeding immediate	None	7 (70%)	10 (100%)	0.067
	Slight	3 (30%)	0	

1st week	None	10 (100%)	10 (100%)	
2nd week	None	10 (100%)	10 (100%)	

## Data Availability

The experimental data used to support the findings of this study are available from the corresponding author upon request after publication 6–12 months.

## Abbreviations

Cm: Centimeter
CO_2_: Carbon dioxide
cp1: Comfort pulse
CW: Continuous wave
GPI: Gingival pigmentation index
Nd: YAG: neodymium-doped yttrium aluminum garnet
nm: Nanometer
ER: YAG: Erbium-doped yttrium aluminium garnet laser
SD: Standard deviation
VAS: Visual analogue scale
W: Watt

## Supplementary Materials

Supplementary Materials Appendix Figure 1. The consort flow chart of patients` eligibility, allocation, and analysis. Appendix Figure 2. Represents CW group treatment of the upper arch. (a) Preoperative. (b) 4th week postoperatively. (c) 3 years postoperatively. Appendix Figure 3. Represents pulsed group treatment of the upper arch. (a) Preoperative view. (b) 4th week postoperatively. (c) 3 years postoperatively.
