# Supplementary material for: Long-Term Esthetically Depigmented Gingiva in a Short Operative Duration, Using Two Modes of 940 nm Diode Lasers—A Randomized Clinical Trial
Source: Int J Dent. 2022 Nov 24;2022:8215348. doi: 10.1155/2022/8215348 (PMC9715332; doi:10.1155/2022/8215348)
Supplement: Supplementary Materials — Appendix Figure 1. The consort flow chart of patients` eligibility, allocation, and analysis. Appendix Figure 2. Represents CW group treatment of the upper arch. (a) Preoperative. (b) 4th week postoperatively. (c) 3 years postoperatively. Appendix Figure 3. Represents pulsed group treatment of the upper arch. (a) Preoperative view. (b) 4th week postoperatively. (c) 3 years postoperatively. [file 8215348.f1.pdf]

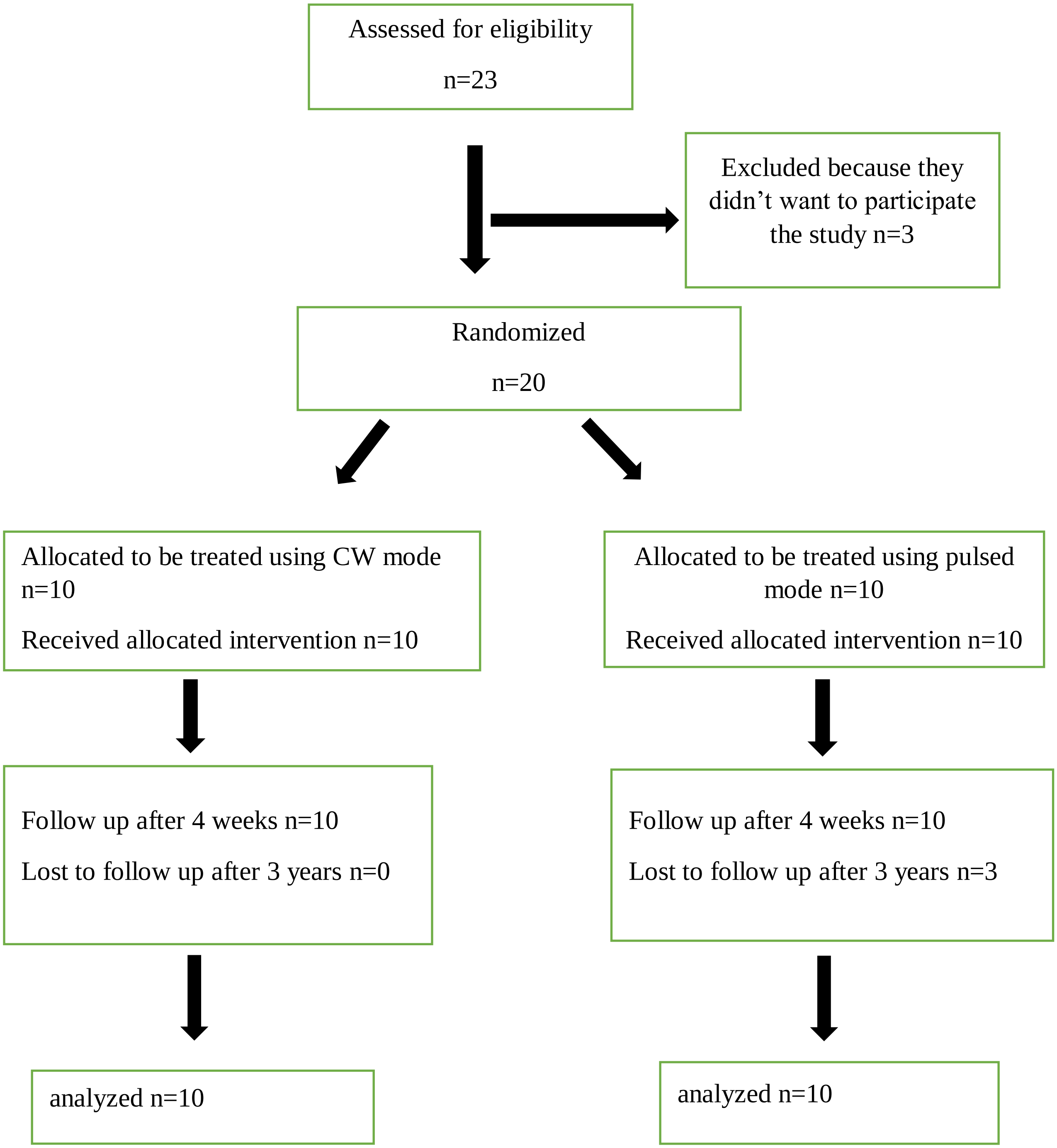

Appendix Figure 1. The consort flow chart of patients` eligibility, allocation and analysis

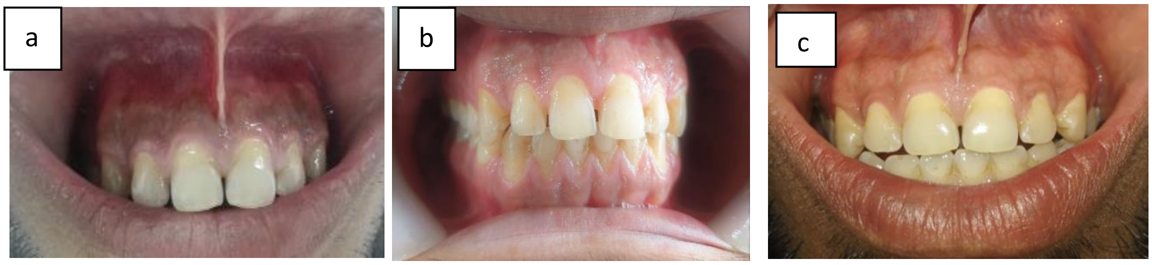

Appendix Figure 2. Represents CW group treatment of upper arch (A) Pre-operative (B) 4th week postoperatively (C) 3 years postoperatively

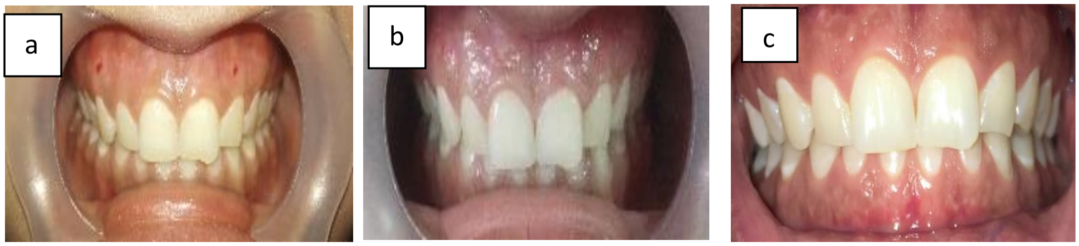

Appendix Figure 3. Represents pulsed group treatment of upper arch (A) Pre-operative view (B) 4th week postoperatively (C) 3 years postoperatively
